# Differential Expression of Aquaporins and Its Diagnostic Utility in Thyroid Cancer

**DOI:** 10.1371/journal.pone.0040770

**Published:** 2012-07-10

**Authors:** Dongfeng Niu, Tetsuo Kondo, Tadao Nakazawa, Tomonori Kawasaki, Tetsu Yamane, Kunio Mochizuki, Yohichiro Kato, Toshiyuki Matsuzaki, Kuniaki Takata, Ryohei Katoh

**Affiliations:** 1 Department of Human Pathology, University of Yamanashi Interdisciplinary Graduate School of Medicine and Engineering, Yamanashi, Japan; 2 Department of Anatomy and Cell Biology, Gunma University Graduate School of Medicine, Maebashi, Japan; 3 Department of Pathology, Tokyo Women’s Medical University Graduate School of Medicine, Tokyo, Japan; Consiglio Nazionale delle Ricerche (CNR), Italy

## Abstract

**Background:**

Aquaporin3 (AQP3) and Aquaporin4 (AQP4) play a major role in transcellular and transepithelial water movement as water channel membrane proteins. Little is known of their expression and significance in human thyroid tissues. Thus, we examined the expression of AQP3 and AQP4 in normal, hyperplastic and neoplastic thyroid tissues in conjunction with human thyroid cancer cell lines.

**Methods and Results:**

Immunohistochemical analyses demonstrated AQP3 in the cytoplasmic membrane of normal C cells, but not in follicular cells. In contrast, AQP4 was not found in C cells but was identified in normal follicular cells. AQP4 was positive in 92% of Graves’ disease thyroids and 97% of multinodular goiters, and we failed to demonstrate AQP3 in these hyperplastic tissues. In neoplastic thyroid lesions, we observed AQP3 in 91% of medullary thyroid carcinomas but in no other follicular cell tumors. AQP4 was demonstrated in 100% of follicular adenomas, 90% of follicular carcinomas, and 85% of papillary carcinomas, while it was negative in all medullary carcinomas and undifferentiated carcinomas. Reverse transcriptase polymerase chain reaction (RT-PCR) analyses revealed AQP3 mRNA expression only in medullary carcinomas and AQP4 mRNA expression in follicular cell-derived tumors except for undifferentiated carcinomas. In thyroid cancer cell lines, using RT-PCR and western blotting, AQP3 mRNA and protein were only identified in the TT cell line (human medullary carcinoma cell line) and AQP4 in the other cell lines. In addition, AQP3 mRNA expression was up-regulated by FBS and calcium administration in both a dose and time dependent manner in TT cells.

**Conclusion:**

The differential expressions of AQP3 and AQP4 may reflect the biological nature and/or function of normal, hyperplastic, and neoplastic thyroid cells and additionally may have value in determining differential diagnoses of thyroid tumors.

## Introduction

Thyroid cancer is the most common malignancy of the endocrine organs, with incidence rates steadily increasing over the last several decades. More than 95% of thyroid carcinomas are derived from follicular cells having a spectrum of differentiation from comparatively indolent carcinomas, including follicular thyroid carcinoma and papillary thyroid carcinoma, to poorly differentiated carcinoma and undifferentiated thyroid carcinoma. Another thyroid carcinoma originating in the thyroid C cell is medullary carcinoma which occurs either sporadically or as part of the inherited, autosomal dominant, multiple endocrine neoplasia (MEN) type 2A and type 2B [1].

The Aquaporins (AQPs) are a family of small (∼30 kDa/monomer) membrane proteins that serve as water channel proteins that play a major role in transcellular and transepithelial water movement [2]–[4]. The AQP family can be divided into 3 subgroups based on their primary sequences: aquaporins (AQP0, AQP1, AQP2, AQP4, AQP5, AQP6 and AQP8) that only transport water, aquaglyceroporins (AQP3, AQP7, AQP9 and AQP10) that are responsible for transporting water, glycerol and other small solutes [5], and superaquaporins belonging to a new subfamily (AQP11 and AQP12) [6], [7].

Aquaporin3 (AQP3) is a typical aquaglyceroporin transporting water, glycerol and urea that plays a major role in fluid homeostasis in normal tissues [8]–[10]. Currently, AQP3 has been demonstrated in many epithelial cells of the urinary, digestive and respiratory tracts [11]–[14], kidney [15]–[17], epidermis [18], [19], eye [20], brain [17], pancreas [21], and prostate [22], In these cell types, high water permeability is associated with a defined physiologic function in reabsorption or secretion. Recently, it has been reported that AQP3 expression may be linked to tumorigenesis and proliferation in carcinomas of several organs such as skin [23], [24], colon [25], kidney [26], and ovary [27].

AQP4 is a transmembrane protein that regulates water entry into and out of specific cells; it is expressed mainly in the kidney and central nervous system including the brain, spinal cord, and optic nerves [28]. The down regulation of AQP4, which has been previously described, results in suppression of cerebral edema in response to water intoxication and stroke which improves clinical indices of survival and neurological status [29], [30]. The high expression level of AQP4 has been reported in glioma [31]. glioblastoma [32], [33], and meningioma [34]. However, the mechanism of AQP4 involvement in brain tumors is still under investigation.

**Table 1:** Primer pairs used in RT-PCR.

Target	Gene accession	Primer sequence	AT (°C)	Product size (bp)
AQP 3	NM_004925	F: 5′-GACAGAAGGAGCTGGTGTCC-3′	58	199
		R: 5**'**-AGAGTGACAGCAAAGCCAAAG-3′		
AQP4	NM_001650	F: 5′-GGTAAGTGTGGACCTTTGTGT-3′	58	203
		R: 5′-CAAAGCAAAGGGAGATGAGAAC-3′		
PGK1	NM_000291	F: 5′-GCTGACAAGTTTGATGAGAAT-3′	58	359
		R: 5′-AGGACTTTACCTTCCAGGAGC-3′		
GAPDH	NM_002046	F: 5′-GATGACATCAAGAAGGTGGTGA-3′	58	186
		R: 5′-TTCGTTGTCATACCAGGAAATG-3′		

F, forward primer; R, reverse primer; bp, base pairs.

To our knowledge, very limited information is available concerning the expression or significance of AQP3 and AQP4 in thyroid tissues. To increase our understanding of this basic biological mechanism in normal and diseased thyroid cells, we examined aquaporin expression (AQP3 and AQP4) in normal, hyperplastic, and neoplastic human thyroid tissues in conjunction with several thyroid cancer cell lines using immunohistochemistry and molecular techniques. We also discuss the usefulness and implications of aquaporin expression for diagnosing and developing treatments for thyroid tumors.

**Table 2:** Summary of immunohistochemistry for AQP3 and AQP4 in thyroid tissues.

Histological type	n	Score of AQP3				Frequency of AQP3 (%)	Score of AQP4				Frequency of AQP4 (%)
		0	1+	2+	3+		0	1+	2+	3+	
Normal thyroid	56	56	0	0	0	0	0	3	37	16	100
Graves’ thyroid	12	12	0	0	0	0	1	2	2	7	92
Multinodular goiter	38	38	0	0	0	0	1	2	21	14	97
Follicular adenoma	20	20	0	0	0	0	0	1	6	13	100
Papillary carcinoma	52	52	0	0	0	0	8	4	24	16	85
Follicular carcinoma	10	10	0	0	0	0	1	1	5	3	90
Undifferentiated carcinoma	10	10	0	0	0	0	10	0	0	0	0
Medullar carcinoma	22	2	1	8	11	91	22	0	0	0	0

0, negative; 1+, focal (1 to 9%); 2+, intermediate (10 to 50%); 3+, diffuse (more than 50%).

**Figure 1:**
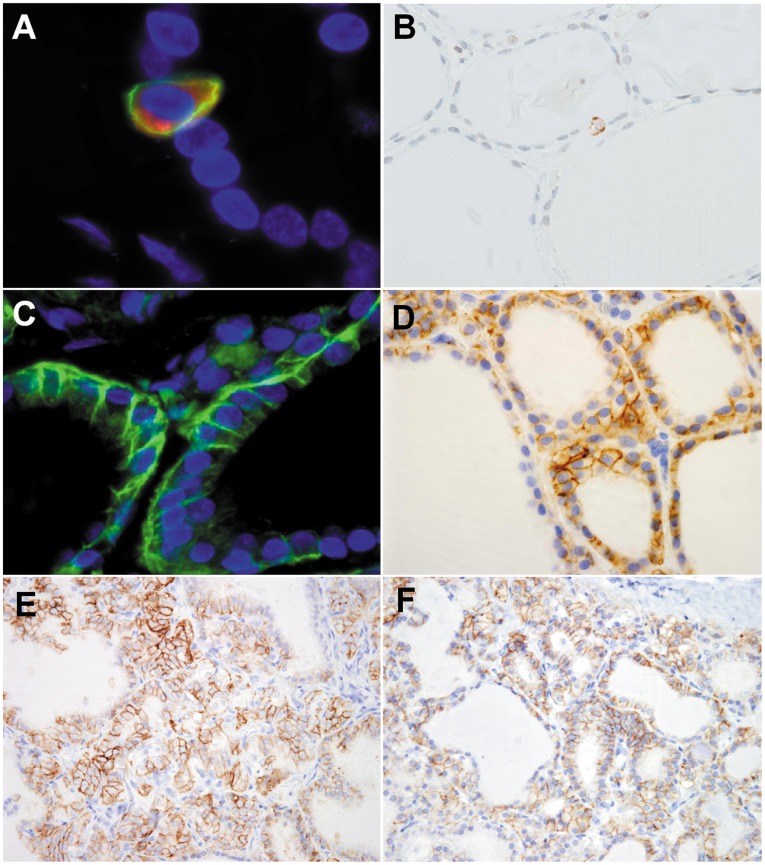
Expression of AQP3 and AQP4 protein in normal and hyperplastic thyroid tissues/cells. (**A**) Immunofluorescence double staining shows AQP3 (FITC: green) expressing in the cytoplasmic membranes of normal human C cells that are positive for calcitonin (TRCR: red), but not in follicular cells. (**B**) Immunoperoxidase staining also shows that AQP3 is positive only in C cells and not in follicular cells. (C) Immunofluorescence analysis exhibits AQP4 protein (FITC: green) in the cytoplasmic membranes of follicular cells. Nuclei are stained by DAPI (blue). (D) AQP4 protein positivity tends to be stronger in tall follicular cells of small follicles. Hyperplastic follicular cells in Graves’ disease (E) and multinodular goiter (F) display prominent positivity of AQP4 in their cytoplasmic membranes. (Magnification: A, C, 1000x; B, D, E, F, 400x).

## Materials and Methods

### Case Selection

We investigated 164 surgical specimens from patients with various thyroid lesions, including 12 cases of Graves’ disease thyroids, 38 multinodular goiters, 20 follicular adenomas, 52 papillary carcinomas, 10 follicular carcinomas, 10 undifferentiated carcinomas, and 22 medullary carcinomas. We also examined normal thyroid tissues adjacent to the tumors. Normal human kidney tissues were obtained from patients who underwent subtotal or total nephrectomy for renal carcinoma. The snap-frozen tissues were stored at −80°C before subsequent isolation of their protein.

**Figure 2:**
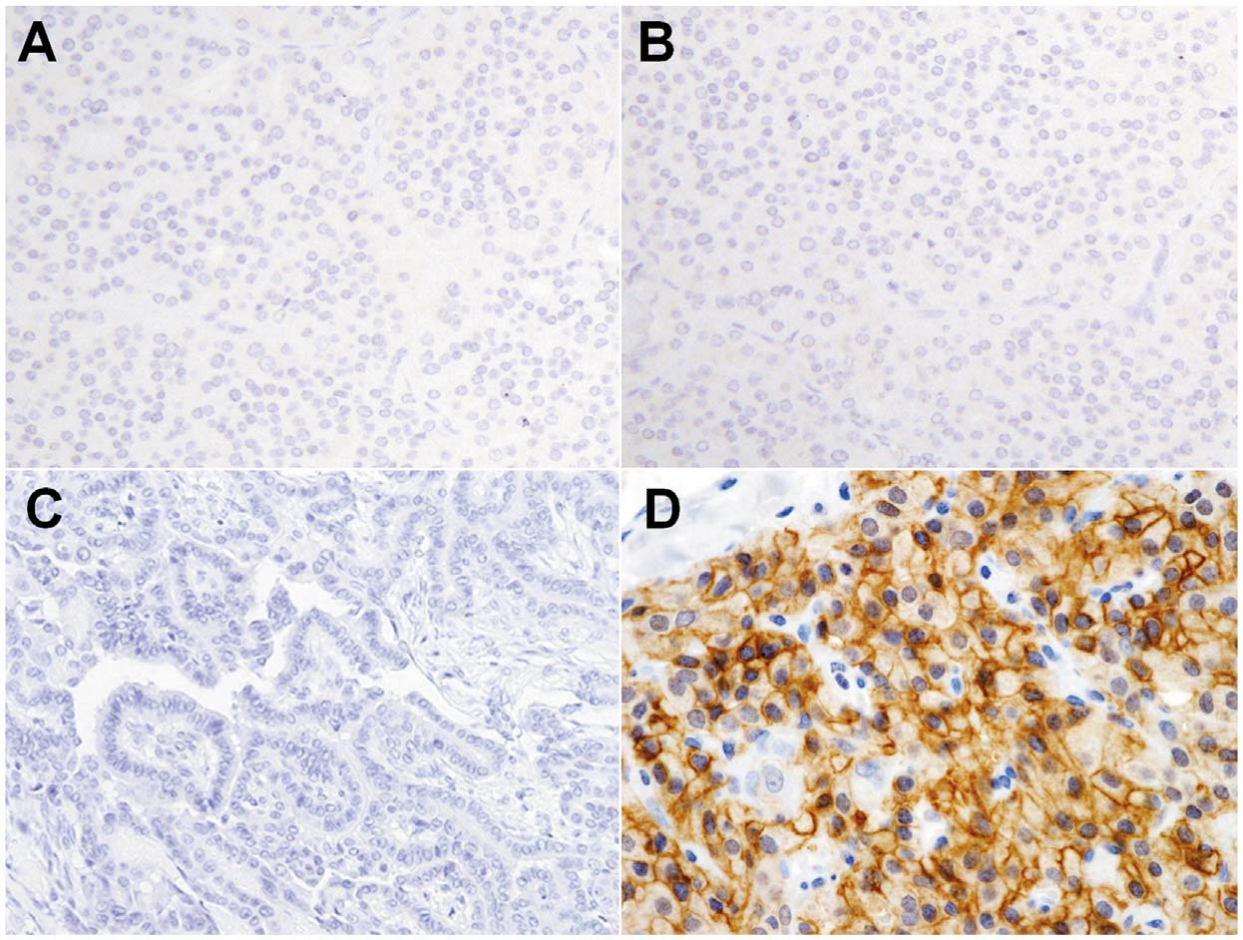
Immunohistochemical staining for AQP3 in neoplastic thyroid tissues. Immunoreactivity of AQP3 is negative in follicular adenoma (**A**), follicular carcinoma (**B**), and papillary carcinoma (**C**). In contrast, AQP3 is clearly immunopositive in the cytoplasmic membranes of medullary carcinoma cells (**D**). (Magnification: 400x).

All slides received hematoxylin-eosin (HE) staining, and the lesions were diagnosed through routine surgical pathological diagnosis on the basis of the World Health Organization classification [1]. The study protocols were approved by the Institutional Ethics Board of the University of Yamanashi.

**Figure 3:**
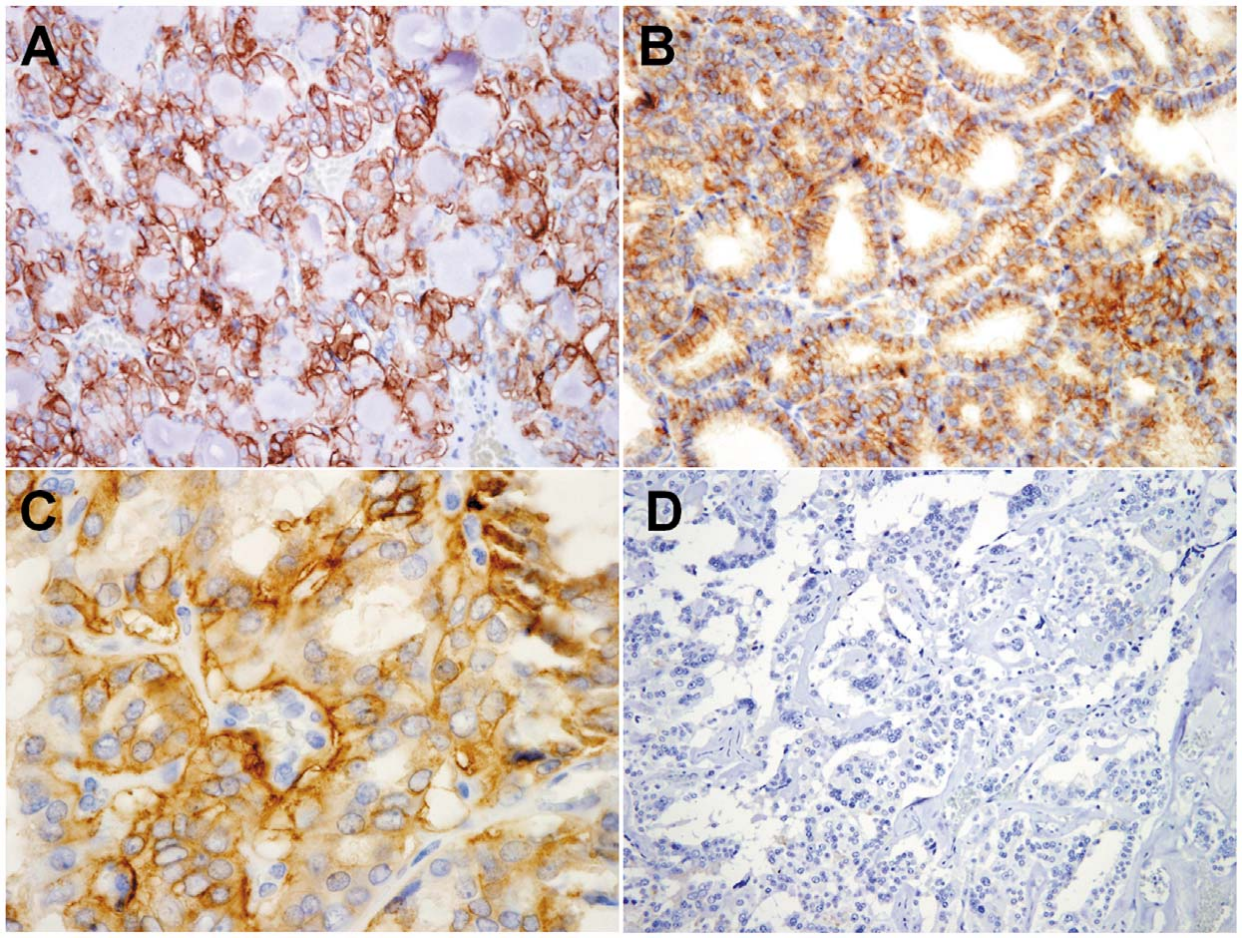
Immunohistochemical staining for AQP4 in human neoplastic thyroid tissues. AQP4 is diffusely immunopositive in neoplastic cells of follicular adenomas (**A**), follicular carcinomas (**B**), and papillary carcinomas (**C**), but negative in medullary carcinoma (**D**). (Magnification: 400x).

### Cell Lines and Cell Culture

We used 8 human thyroid carcinoma-derived cell lines: KTC-1, TPC-1, WRO, 8305C, 8503C and TT have been previously described [35], [36], and UA-1 and UA-2 were originally established cell lines derived from human undifferentiated carcinoma [37]. Cells were maintained in RPMI 1640 (GIBCO, Grand Island, NY, USA) and supplemented with 10% fetal bovine serum (FBS), streptomycin sulfate (100 mg/L), and penicillin G sodium (100 mg/L). Cells were cultured in a standard humidified incubator at 37°C in a 5% CO_2_ atmosphere.

**Figure 4:**
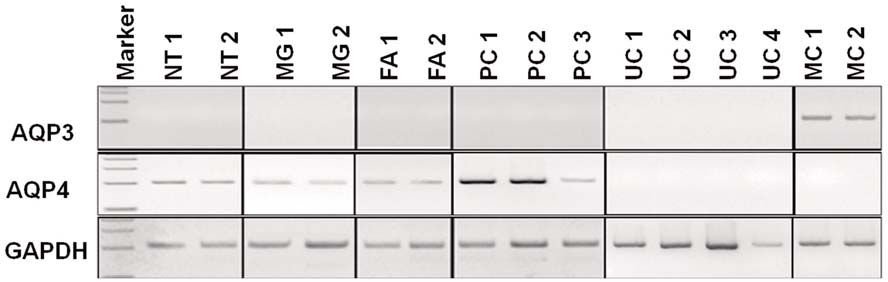
Expression of AQP3 and AQP4 mRNA in human normal, hyperplastic and neoplastic thyroid tissues. RT-PCR shows AQP3 mRNA only in medullary carcinomas (MC), and absent in normal thyroid tissues (NT), multinodular goiters (MG), follicular adenomas (FA), papillary carcinomas (PC), and undifferentiated carcinomas (UC). In contrast, AQP4 mRNA is demonstrated in NT, MG, FA, and PC, and negative in all UC and MC.

The TT cells were seeded in a 10 cm dish until 80% confluence and after serum starvation for 24 hours. They were then treated in medium with graded concentrations of FBS (0%, 1%, 5% and 10%) for 24 hours. The cells were also cultured in medium with 5% FBS for varying times of 0, 4, 8, 12, 16, 24 and 36 hours. We treated the TT cell line with a series of calcium concentrations (0, 0.1, 1 and 10 uM) (Wako, Tokyo, Japan) and also with 10 uM of calcium for 0, 4, 8, 12, 16, and 24 hours.

**Figure 5:**
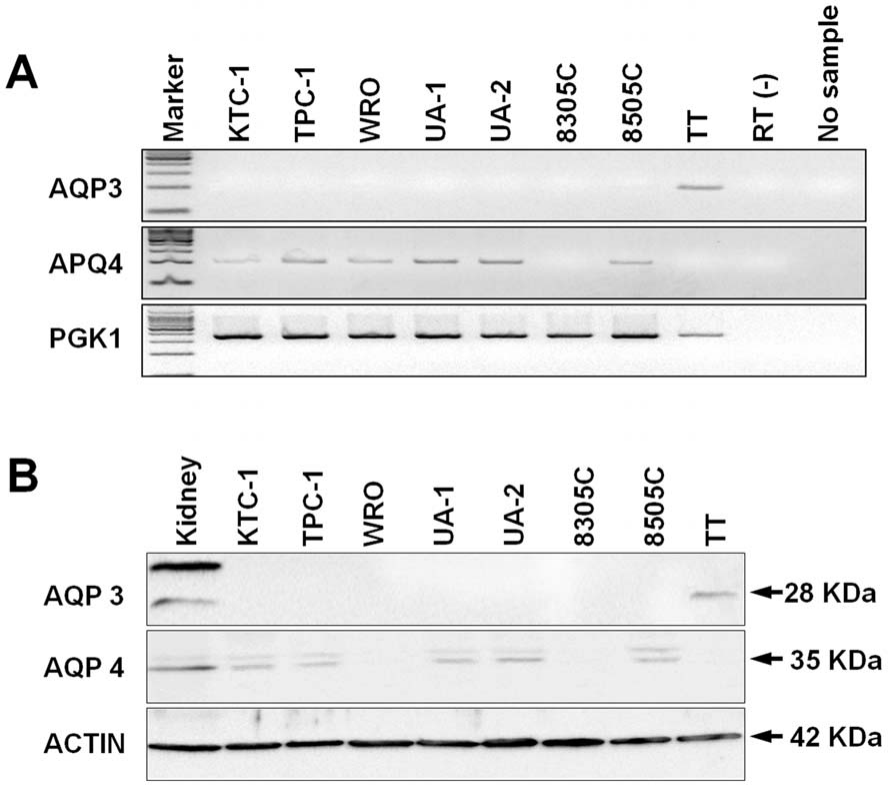
Expression profiles of AQP3 and AQP4 in thyroid carcinoma cell lines. (A) RT-PCR analysis shows that AQP3 mRNA is specifically identified in the TT cell line, whereas AQP4 is detected in the follicular cell-derived lines (KTC-1, TPC-1, WRO, UA-1, UA-2, and 8505C). (**B**) Western blotting confirms that AQP3 protein is seen in the TT cell line and not in the other cell lines. AQP4 protein is identified in five cell lines (KTC-1, TPC-1, UA-1, UA-2, and 8505C), while absent in three cell lines (TT, 8305C and WRO).

### Immunohistochemistry

We performed immunohistochemical staining on 3-µm sections of formalin-fixed and paraffin-embedded tissues. A detailed protocol has been described previously [37], [38]. Briefly, deparaffinized sections were incubated with the affinity-purified rabbit polyclonal antibody to rat/human AQP3 at 1∶500 dilution [39] or the affinity-purified rabbit polyclonal antibody to human AQP4 at 1∶1000 dilution (Sigma, St. Louis, USA) for 1 hour at room temperature. After rinsing 3 times with PBS, the sections were incubated with the labeled polymer (Envision/horseradish peroxidase, DAKO, Glostrup, Denmark) for 1 hour at room temperature. We used normal cells/tissue from the collecting duct of the kidney as a positive control and omitted the primary antibody for the negative control. The amounts of immunoreactivity of AQP3 and AQP4 were evaluated using a grading scale from 0 to 3+: 0, no immunoreactivity; 1+, focal immunoreactivity (1% to 9% positive cells); 2+, intermediate immunoreactivity between 1+ and 3+; 3+, diffuse immunoreactivity (more than 50% positive cells).

**Figure 6:**
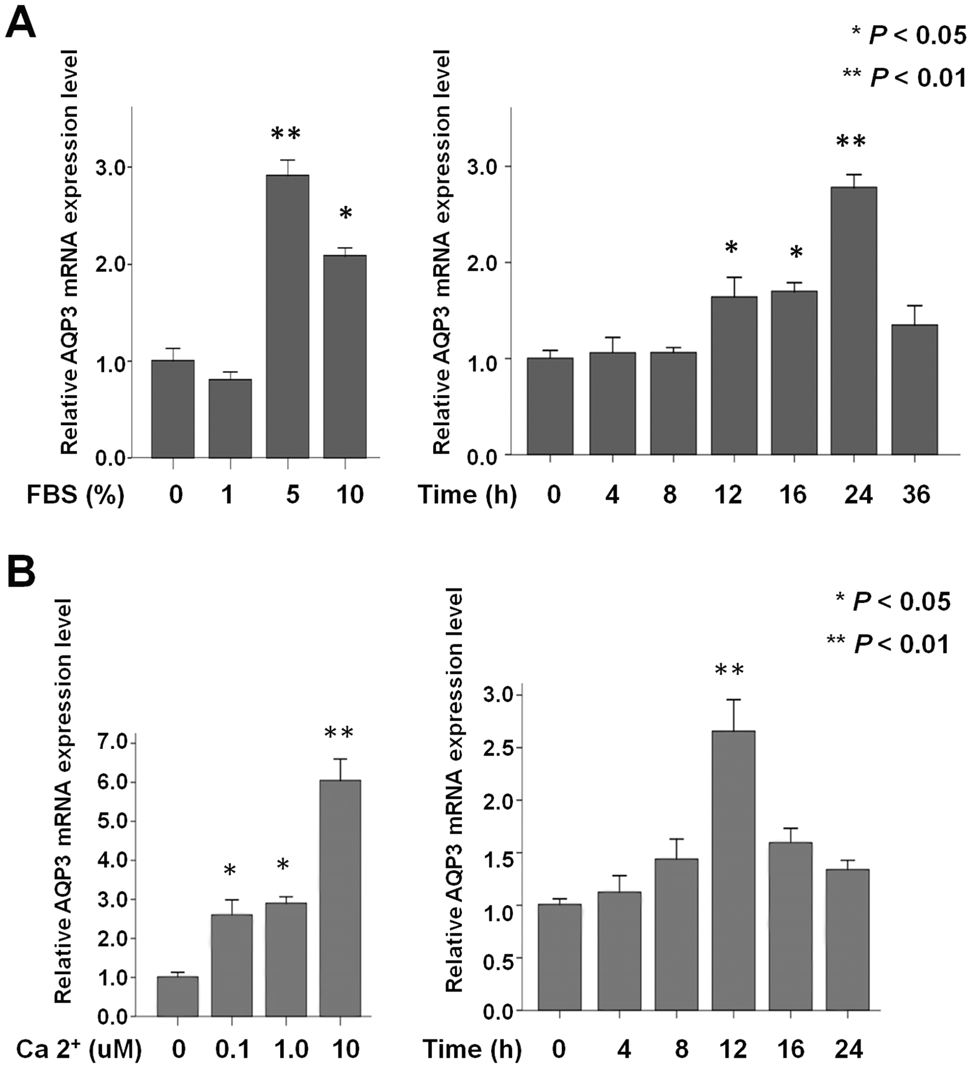
Real time RT-PCR analysis for AQP3 mRNA in the TT cell line stimulated by fetal bovine serum (FBS) and calcium. (**A**) AQP3 mRNA level is significantly increased by 5% and 10% FBS treatments. (**B**) AQP3 mRNA level is significantly up-regulated by calcium administration in a dose-dependent manner. Error bars, standard deviation. Significant difference, *P<0.05, **P<0.01.

### Immunohistofluorescence

We also carried out double immunohistofluorescence on 3-µm sections of formalin-fixed and paraffin-embedded tissues through a sequential approach for primary antibodies raised from the same species. The treated sections were incubated with the first primary antibody of rabbit polyclonal antibody to rat/human AQP3 at 1∶500 dilution at room temperature for 1 hour, then with swine anti-rabbit fluorescein isothiocyanate (FITC) at 1∶20 dilution (DAKO, Tokyo, Japan) at room temperature for 1 hour. After rinsing 3 times, the sections were re-fixed in formalin at room temperature for 1 hour and incubated with rabbit polyclonal antibody to human calcitonin (DAKO, CA, US) at 1∶5000 dilution at room temperature for 1 hour, then with anti-rabbit fluorescein Rhodamine at 1∶40 dilution (DAKO, Tokyo, Japan) at room temperature for 1 hour. Nuclei were counterstained with 4′, 6-diamiono-2-phenylindole (DAPI). We visualized fluorescence using an epi-illuminant fluorescence microscope (BX50, Olympus, Tokyo, Japan).

### Protein Isolation and Western Blotting

Culture cells and kidney tissues were lysed in a polytron homogenizer in a radioimmunoprecipitation assay (RIPA) lysis buffer with proteinase inhibitors (1× phosphate-buffered saline (PBS), 1% Nonidet P40, 0.5% sodium deoxycholate, 0.1% sodium dodecyl sulfate (SDS), 1 mmol/L phenylmethylsulfonyl fluoride (PMSF), 12 µg/mL aprotinin, and 1 mmol/L sodium orthovanadate).

Equal amounts of protein (30 µg) solubilized in sample buffer were separated on 10% SDS polyacrylamide gels and transferred electrophoretically to polyvinylidene difluoride (PVDF) membranes. Membranes were blocked in Tris-buffered saline (TBS) containing 0.05% Tween 20 plus 5% nonfat dried milk for 1 hour at room temperature and probed with primary antibodies at 4°C overnight.

Primary antibodies were used at the specified dilutions: rabbit anti-AQP3 polyclonal antibody at 1∶2000 (Santa Cruz Biotechnology, Santa Cruz, USA) and the affinity-purified rabbit polyclonal antibody to human AQP4 at 1∶2000 (Sigma, St. Louis, USA) and mouse monoclonal to beta actin at 1∶5000 dilution (Abcam, Cambridge, UK). Membranes were washed three times for 10 min each in TBS containing 0.05% Tween-20 and incubated with horseradish peroxidase (HRP)–conjugated goat anti-rabbit or anti-mouse secondary antibody at 1∶2000 dilution (Santa Cruz Biotechnology, Santa Cruz, USA) for 1 hour at room temperature. Targeted proteins were visualized using an enhanced chemiluminescence detection system (Amersham, Buckinghamshire, UK).

### RNA Extraction, cDNA Synthesis, RT-PCR and Quantitative Real-time PCR

We isolated total RNA of thyroid tissues from 10-µm sections of formalin-fixed and paraffin-embedded tissues using Recover All™ Total Nucleic Acid Isolation Kit (Applied Biosystems, New Jersey, USA) according to the manufacturer’s instructions. Total RNA of cultured cells was isolated using TRIzol (Invitrogen, Carlsbad, USA), and cDNA was generated using the TaqMan reverse transcription (RT) reagent kit (Applied Biosystems, New Jersey, USA). Specific PCR primers targeted for AQP3, AQP4, glyceraldehyde-3-phosphate dehydrogenase (GAPDH) and PGK1 were designed as listed in Table 1. GAPDH [40] and PGK1 [41] were used as internal controls. Amplification was done using HotStarTaq DNA polymerase kit (Qiagen, Tokyo, Japan). PCR conditions were as follows: (1) 95°C for 15 min; (2) 30 cycles of 94°C for 30 s, 56°C or 58°C for 30 s, and 72°C for 1 min; (3) 72°C for 10 min; and (4) 4°C hold. Negative controls omitting RT or without cDNA and appropriate positive controls were included in each PCR reaction.

Real-time PCR was carried out with MyiQ™ Single-Color Real-time PCR detection system (Applied Biosystems, CA, USA). Approximately 2.5 µl of cDNA was amplified in each 25 µl of PCR reaction mix containing 12.5 µl of 2X SYBR Green Master Mix (Qiagen) plus 0.75 µl each of 10 mmol forward and reverse primers and 8.5 µl DEPC treated water. Total reaction volume was 25 µl. We started amplification at 95°C for 15 min as the first step, followed successively by 45 cycles of PCR at 95°C for 30 s, 58°C for 30 s and 72°C for 60 s. The amount of mRNA of the target gene was normalized to that of GAPDH using the ΔCt method, as described in the manufacturer’s protocol.

### Statistics

Data are presented as mean ± SD. Statistical significance was set at *p*<0.05. Data analyses were performed using SPSS version 13.0 for windows (SPSS Inc., Tokyo, Japan).

## Results

### AQP3 and AQP4 Protein Expression in Thyroid Tissues

The immunohistochemical results of AQP3 and AQP4 using human thyroid tissues are presented in Table 2. On immunohistochemical examinations, we found AQP3 protein in the cytoplasmic membrane of normal thyroid C cells which were positive for calcitonin, while it was completely negative in follicular cells (Fig. 1A and B). We also failed to demonstrated immunopositivity of AQP3 in hyperplastic follicular cells of Graves’ disease thyroids and multinodular goiters.

In contrast, AQP4 was positive in the cytoplasmic membrane of normal thyroid follicular cells and negative in C cells (Fig. 1C). AQP4 tended to be positive in tall follicular cells of small follicles and negative in flat follicular cells of large follicles (Fig. 1D). Hyperplastic follicular cells in most of the Graves’ disease thyroids and multinodular goiters were positive for AQP4 in their cytoplasmic membranes; the positive frequency was 92% in Graves’ disease thyroids (Fig. 1E) and 97% in multinodular goiters (Fig. 1F).

In neoplastic thyroid tissues, immunoreactivity of AQP3 was frequently demonstrated in the cytoplasmic membrane of medullary carcinoma cells (Fig. 2D). The overall positive frequency of AQP3 in medullary carcinomas was 91% (20/22). Among 20 medullary carcinomas positive for AQP3, the distribution patterns of AQP3-positive cells varied: 55% (11/20) in diffuse, 40% (8/20) in intermediate, and 5% (1/20) in focal medullary carcinomas. AQP3 was not identified in the follicular cell-derived tumors (Fig. 2A–C).

In contrast, we frequently observed AQP4 in the cells of follicular adenomas (100% of cases), follicular carcinomas (90% of cases), and papillary carcinomas (85% of cases) (Fig. 3A–C). Most of AQP4-positive, neoplastic tissues exhibited intermediate or diffuse positive cell distribution. We did not observe AQP4 in the cells of undifferentiated carcinomas or medullary carcinomas (Fig. 3D).

### AQP3 and AQP4 mRNA Expression in Thyroid Tissues

As shown in Figure 4, RT-PCR analyses using oligonucleotide primers revealed that all normal and hyperplastic thyroid specimens examined expressed a band suggestive of AQP4, whereas AQP3 was not identified.

In neoplastic thyroid tissues, we identified AQP3 mRNA expression only in medullary carcinomas. We identified AQP4 mRNA expression in follicular adenomas and papillary carcinomas but not in undifferentiated carcinomas or medullary carcinomas. These results support the results of our immunohistochemical analyses.

### AQP3 and AQP4 Expression in Thyroid Carcinoma Cell Lines

We examined mRNA expression profiles of AQP3 and AQP4 in eight thyroid carcinoma cell lines using RT-PCR (Fig. 5A). We specifically observed expression of AQP3 mRNA in the TT cell line which was established from human medullary carcinoma. In contrast, we saw AQP4 mRNA expression in KTC-1, TPC-1, WRO, UA-1, UA-2, and 8505C cell lines but not in the TT and 8305C cell lines.

On western blotting analyses, we detected AQP3 protein only in the TT cell line but no other cell lines, whereas, AQP4 protein was identified in the cell lines of KTC-1, TPC-1, UA-1, UA-2, and 8505C but not in TT. Neither AQP3 nor AQP4 was observed in the WRO or 8305C cell lines (Fig. 5B).

### Fetal Bovine Serum (FBS) and Calcium Regulates AQP3 mRNA Expression in TT Cells

Having determined that AQP3 is specifically detected in TT cells, we examined the stimulating effect of varying concentrations of the FBS medium on AQP3 mRNA levels using a series of FBS concentrations (0%, 1%, 5% and 10%) over 24 hours (Fig. 6A). AQP3 mRNA levels were significantly increased in 5% and 10% FBS treatments compared to no FBS treatment (*P*<0.01).

Subsequently, we examined the effect of stimulation time (0, 4, 8, 12, 16, 24, and 36 hours) on the TT cells cultured with 5% FBS. AQP3 mRNA levels at 12, 16 and 24 hours were significantly increased (*P*<0.05 vs. control of 0 hour), reaching a peak at 24 hours (*P*<0.01).

As shown in Figure 6B, the cells were treated with different doses of calcium (0, 0.1, 1, 10 and 100 uM). AQP3 mRNA expression was significantly up-regulated by calcium administration in a dose-dependent manner compared to the control (*P*<0.05). We observed the highest AQP3 mRNA expression in 10 uM of calcium, representing a 6-fold increase compared to the control (0 uM calcium).

We examined the effect of stimulation times (0, 4, 8, 12, 16, and 24 hours) with a 10 uM calcium treatment in the TT cells and found a significant increase in AQP3 mRNA expression with time; it reached a peak at 12 hours (*P*<0.01).

## Discussion

AQP3 is a membrane transporter of water and glycerol expressed in plasma membranes of cells to maintain a balance of fluid metabolism. AQP3 expression has been reported in various organs of rats and mice [11], [12]. In normal tissues/cells of humans, Mobasheri et al. [42] reported a strong expression of AQP3 in basolateral membranes of the distal nephron (medullary collecting ducts), distal colon, upper airway epithelium, transitional epithelium of the urinary bladder, tracheal, bronchial and nasopharyngeal epithelium, and stratified squamous epithelial cells of the esophagus and anus. Our recent immunohistochemical study using a large series of human tissues revealed additional cells/tissues producing AQP3 such as pituitary cells, thymic epithelial cells, fundic gland cells of the stomach, islet cells of the pancreas, spermatogenic cells of the testis, sudoriferous gland cells, sebaceous gland cells, and apocrine gland cells of the skin [39].

AQP4 is one of the water-selective isoforms in the AQP family. This water channel protein is reported to be mainly expressed in brain tissue including the glia limitans, ependymal lining, cerebellum, hippocampal denate gyrus, and in the supraoptic and paraventricular nuclei of the hypothalamus [28]. In addition, low but significant AQP4 expression also has been found in the neocortex, hippocampal areas CA1-CA3, nucleus of the stria terminals, and the medial habenular nucleus [31]–[33].

To our knowledge, AQPs’ expression in the thyroid has not been reported up to this point. In this current study using immunohistochemistry, we demonstrated the expression of AQP3 and AQP4 in normal human thyroid tissues. Interestingly, AQP3 was strictly expressed in C cells, demonstrated by double staining for AQP3 and calcitonin. In contrast, AQP4 expression was demonstrated in follicular cells but not in C cells. It is generally known that C cells producing calcitonin and follicular cells producing T3 and T4 are derived from different origins; C cells originate from cells associated with the fourth or fifth pharyngeal pouch, which contributes to the ultimobranchial body and follicular cells are mostly derived from the endoderm at the base of the tongue. Therefore, it is conceivable that the differential expression of AQP3 in C cells and follicular cells could be associated with their developmental origin and/or function.

In hyperplastic thyroid tissues such as thyroids with Graves’ disease or multinodular goiters, our immunohistochemical analyses revealed the AQP4 protein in most of the tissues: a positive frequency of 92% in Graves’ disease thyroids and 97% in multinodular goiters. We failed to demonstrate AQP3 protein and mRNA in the hyperplastic tissues. These findings suggest that AQP4 may play an important role in fluid homeostasis in hyperplastic follicular cells as well as normal follicular cells.

Our recent paper described diffuse immunoreactivity of AQP3 in a wide variety of human tissues such as squamous cell carcinoma of skin, esophagus and endometrial cervix, urothelial carcinoma, salivary carcinoma, etc. Medullary thyroid carcinomas have also been reported as positive for AQP3 [39]. In this study, we demonstrated that AQP3 was positive in most medullary thyroid carcinomas and negative in follicular cell-derived tumors using immunohistochemistry in conjunction with RT-PCR. In addition, we found AQP3 expression in the TT cell line which originates from human medullary carcinoma. Therefore, we suggest that AQP3 may be associated with the biological nature of human medullary thyroid carcinoma cells.

Based on this specific expression of AQP3 in TT cells, we demonstrated that AQP3 mRNA levels increased significantly with FBS treatment. FBS is a common component of animal cell culture media; several tumor cell types are stimulated using FBS [43], [44]. This suggested to us that certain components in FBS are involved in regulating AQP3 expression, but the exact biological molecular mechanism must still be clarified. We also found that AQP3 mRNA was induced by calcium in a dose-dependent manner in TT cells. Calcium homeostasis in vivo is regulated by both calcitonin and parathyroid hormone [45]. Our data suggest that AQP3 might be involved with calcium metabolism in medullary thyroid carcinoma cells.

AQP4 expression has been reported in a series of brain tumors such as glioma, glioblastoma and astrocytoma [31]–[33]. Our study is the first report showing that AQP4 expresses in thyroid tumors; follicular cell-derived tumors, except for undifferentiated carcinoma, frequently produce AQP4. The positive frequency of AQP4 was 100% in follicular adenomas, 90% in follicular carcinomas, and 85% in papillary carcinomas. Our RT-PCR results for AQP4 mRNA entirely support these immunohistochemical analyses; we identified AQP4 mRNA in follicular cell-derived carcinoma cell lines. Therefore, we suggest that AQP4’s water metabolism mechanism is well maintained during neoplastic transformation. As to the role of AQPs in human tumors, several investigators have reported that AQPs can relate to migration, invasion and proliferation of tumor cells [46]–[49]. From this point of view, it is conceivable that the expression of AQP3 and/or AQP4 may contribute the biological behavior of thyroid tumors.

We should also consider the possibility of other AQPs in undifferentiated thyroid carcinoma in which neither AQP3 nor AQP4 was expressed. These findings give us clues that other AQP subtypes may be involved in water movement in undifferentiated thyroid carcinomas, and further study is encouraged.

In conclusion, our findings suggest that the differential expressions of AQP3 and AQP4 may reflect the biological nature and/or function of normal, hyperplastic, and neoplastic thyroid cells and, additionally, have some value for diagnosing thyroid tumors.
